# Supplementary figures and images for: Systematic Assessment of Small RNA Profiling in Human Extracellular Vesicles
Source: Cancers (Basel). 2023 Jun 30;15(13):3446. doi: 10.3390/cancers15133446 (PMC10340377; doi:10.3390/cancers15133446)

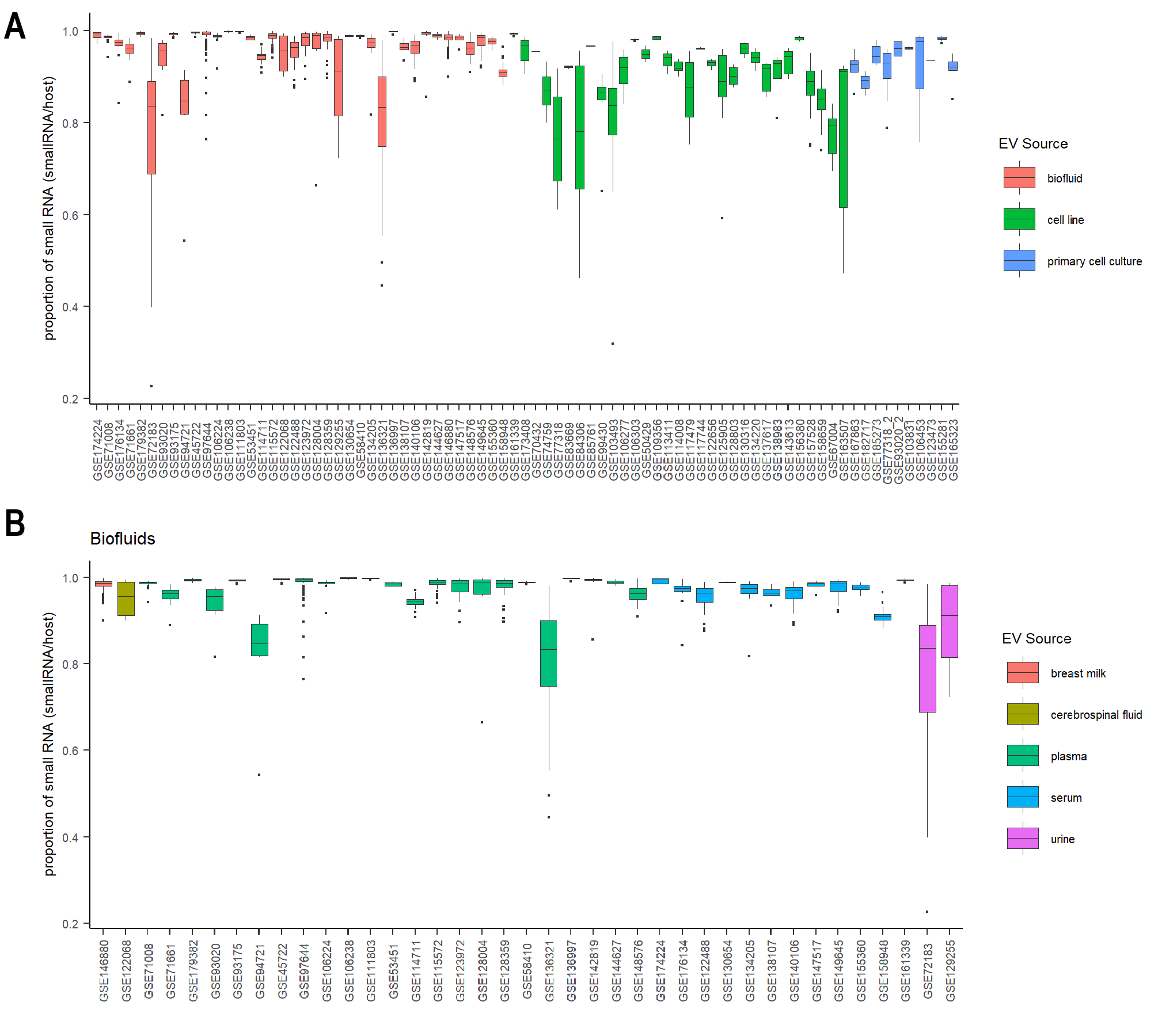

Supplement: Supplementary file 1 [file cancers-15-03446-s001.zip › Supplementary Files/FigS1.tif]

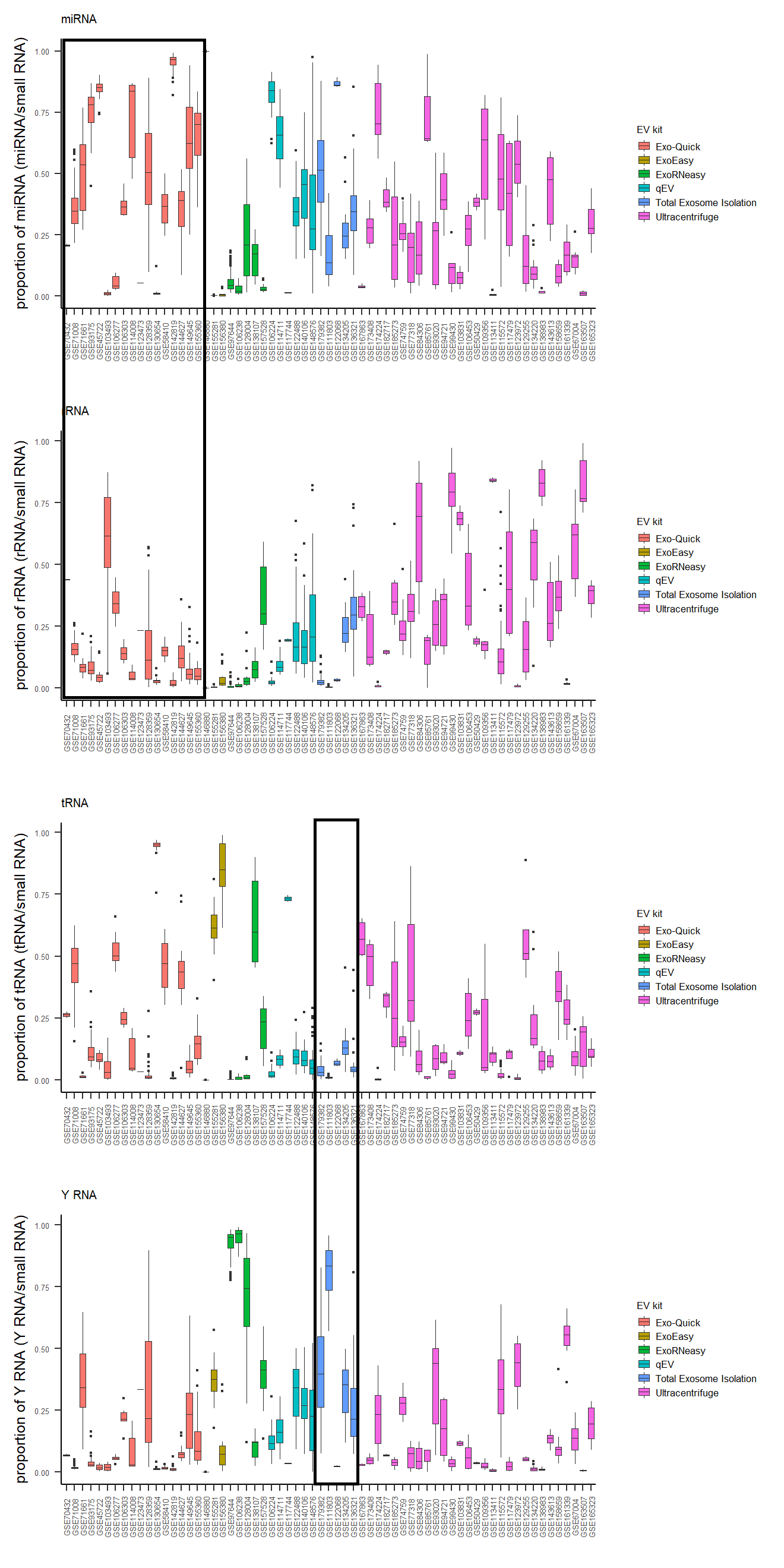

Supplement: Supplementary file 1 [file cancers-15-03446-s001.zip › Supplementary Files/FigS2.tif]

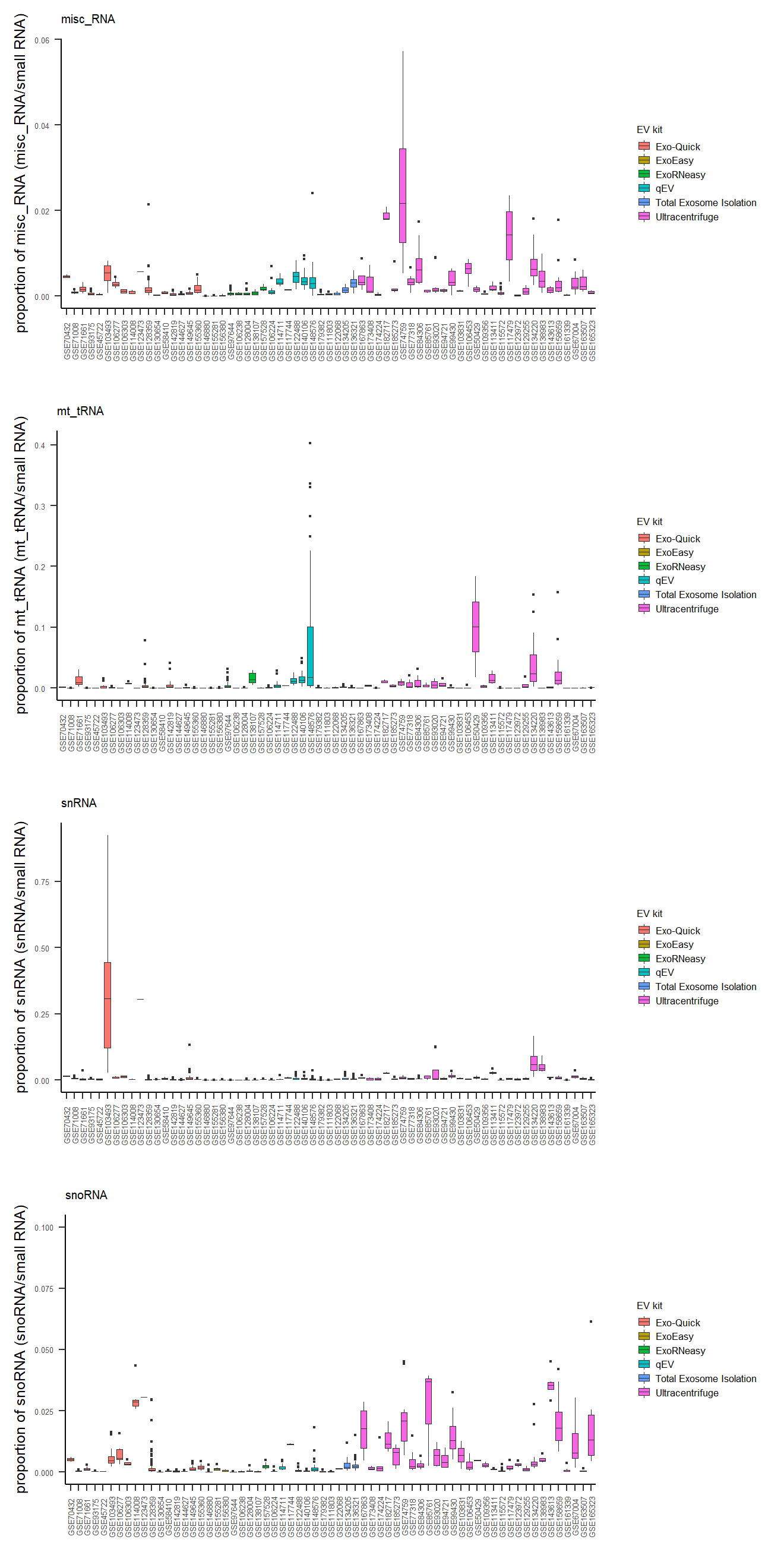

Supplement: Supplementary file 1 [file cancers-15-03446-s001.zip › Supplementary Files/FigS3.tif]
